# Comparing Endodontic Treatment Prevalence in Diabetes Mellitus and Non-Diabetic Periodontitis Patients: A Retrospective Case-Control Investigation

**DOI:** 10.1016/j.identj.2025.100846

**Published:** 2025-07-08

**Authors:** L.P.M. Weijdijk, D.E. Slot, M. el Kadi, G.A. Van der Weijden

**Affiliations:** a Department of Periodontology Academic Center for Dentistry Amsterdam (ACTA), ACTA is a joint venture between the Faculty of Dentistry of the University of Amsterdam and the Faculty of Dentistry of the Vrije Universiteit Amsterdam, Amsterdam, The Netherlands; b Department of Oral and Maxillofacial Surgery, Amsterdam UMC and Academic Centre for Dentistry Amsterdam (ACTA), ACTA is a joint venture between the Faculty of Dentistry of the University of Amsterdam and the Faculty of Dentistry of the Vrije Universiteit Amsterdam, Amsterdam, The Netherlands; c The Clinic for Periodontology, Utrecht, The Netherlands

**Keywords:** Diabetes mellitus, Root canal treatment, Endodontic treatment, Tooth loss, Dental caries, Periodontitis

## Abstract

**Objective:**

Apply a case-control retrospective analysis to assess possible differences in the prevalence of endodontically treated teeth in patients with diabetes(DM) and non-diabetics(NDM).

**Methods:**

A convenience sample of DM and NDM adults diagnosed with periodontitis and referred to a clinic specializing in periodontal therapy were matched based on age, gender, and year of intake. To assess the number of endodontically treated teeth, a full-mouth set of radiographs was required. Every root-filled tooth was recorded. Sub-analyses were conducted to assess the distribution of teeth based on tooth type and their location in the upper and lower jaws. Additionally, the number of teeth present was counted. Relationships between endodontically treated teeth and related variables were analysed.

**Results:**

Total 233 periodontitis patients with DM were found to be eligible for inclusion and accordingly matched to 233 periodontitis patients without DM. Between DM and NDM, no statistically significant differences were found in the mean percentage of endodontically treated teeth (DM: 6.88%; NDM: 7.34%; *P =* .60), tooth type, or jaw type, nor in the average number of teeth (DM: 25.2; NDM: 25.3; *P =* .68). Based on the multivariate analyses with correction for age, smoking status, and number of teeth, DM was not significantly associated with the number of endodontically treated teeth (OR = 1.16;95% CI: [0.79;1.70]; *P =* .46).

**Conclusion:**

In matched patient sample with adult periodontitis, there was no significant association between DM appearance and the number of endodontically treated teeth or tooth loss when compared with NDM.

**Clinical relevance:**

This suggests that in periodontitis patients DM appears not to be a risk factor influencing the degree of tooth decay necessitating endodontic intervention, nor does it seem to contribute to an increased likelihood of tooth loss in this context.

## Introduction

The World Health Organization (WHO) classifies diabetes mellitus (DM) as a significant noncommunicable disease (NCD), responsible for an estimated 1.6 million deaths annually.^1^ DM is a complex chronic condition characterized by defects in insulin secretion or acquired insulin resistance within the human body.^2^ Due to aging populations and lifestyle factors, DM represents a growing global public health concern, expected to place increasing demands on healthcare systems in the coming decades.^3^ Over the years, substantial scientific evidence has established associations between systemic health and oral health, with DM frequently highlighted as a key systemic disorder influencing oral conditions.^4^

According to the Global Burden of Disease Study, dental caries and periodontal diseases are prominent chronic NCDs that necessitate prevention and treatment.^5^^,^^6^ Evidence suggests that individuals with DM have an elevated risk of developing both dental caries^7^^,^^8^ and periodontitis,^9^ the two primary causes of tooth loss.^10^ Research indicates that patients with DM exhibit a modest but significantly higher risk of tooth loss compared to non-diabetics (NDM) individuals.^11^ Furthermore, data from 54,936 root canal treatments demonstrated a significant association between DM and an increased frequency of non-retained root-filled teeth.^12^

The biological process of periapical healing following endodontic treatment is found to be impaired in patients with DM, as shown by recent studies reporting negative impacts on periapical healing outcomes in DM populations compared to NDM controls.^13^, ^14^, ^15^, ^16^ This impairment may be attributed to mechanisms such as altered immune responses, cytokine dysregulation, oxidative stress, and impaired neutrophil function.^17^ Additionally, DM is associated with heightened inflammation and tissue degeneration, particularly after dental interventions.^18^ Hyperglycaemia has been implicated in predisposing individuals to dental pulp inflammation and necrosis, which may subsequently increase the need for root canal treatments.^19^^,^^20^

Apical periodontitis (AP), a pathophysiological inflammatory condition primarily caused by microbial infections,^21^ often arises in necrotic or previously treated pulp tissue. In some cases, infections extend into peri-radicular tissues, resulting in acute or chronic abscesses.^21^ Emerging evidence indicates a bidirectional relationship between AP and DM.^22^ For instance, a Brazilian study identified significant associations between AP and DM or prediabetes in a rural population.^23^ Conversely, AP has also been linked to an increased risk of DM.^24^ These findings underscore the need for tailored clinical management of DM patients. Notably, a retrospective cohort study revealed that patients with periodontal disease also exhibit a higher risk of developing AP in endodontically treated teeth compared to those without periodontal disease.^25^

Given the elevated risk of AP in diabetic patients,^25^ it is of interest to investigate whether this predisposition translates into a higher prevalence of endodontic treatments. This is particularly relevant as asymptomatic tooth infections are common and may go unnoticed, and untreated.^26^ Moreover, inadequately managed caries in DM patients – who are reported to be more susceptible to tooth decay^27^ – can progress into deeper lesions, ultimately leading to pulp necrosis.^28^ A recent systematic review (SR) reported a marginally significant finding of nearly double the prevalence of root canal treatments in DM patients compared to NDM controls.^29^ Similarly, a cross-sectional study observed a higher number of endodontically treated teeth in DM individuals compared to NDM.^30^

However, the certainty of evidence remains low due to heterogeneity among studies. The present study aimed to evaluate in a matched patient group with periodontitis the prevalence of endodontically treated teeth, comparing those with DM to those without.

## Methods

### Design and ethics

This study was prepared according to the guidelines suggested by the STROBE and RECORD checklists (see Online Appendix S1 and S2). These checklists provide recommendations of items to include in reports of observational studies.^31^, ^32^, ^33^ Approval by a Review Board for Human Research was not required.^34^ The institutional review board of the Academic Centre for Dentistry Amsterdam (ACTA) approved the protocol under reference number 2021-11526 (see Online Appendix S3).

### Data set of the studied population

The patient population was a convenience sample from the Clinic for Periodontology in the city of Utrecht, the Netherlands, which is a private clinic specialized in- and restricted to periodontology. The rationale for selecting this study population was that for periodontitis patients usually a full set of radiographs is available for diagnosis and treatment planning.^35^^,^^36^ Also, the prevalence of DM was considered to be higher among periodontitis patients than among a random patient sample.^9^ Patient record files were available from the Dental Practice Management Software Package Simplex (Gé Systems, the Netherlands B.V.) and radiographs from the image analysis software VisiQuick (Citodent Imaging B.V., Amsterdam, the Netherlands). The dataset was comprehensively compiled through a retrospective analysis of the medical records encompassing all patients who received treatment between 2003 and 2019. The included patients had given their approval for their medical records to be used for scientific and quality evaluation purposes by signing an informed consent form prior to the intake appointment.

### Diagnosis, status, and case selection

#### Periodontal diagnosis

All included patients were diagnosed with moderate to severe adult periodontitis based on the criteria proposed by van der Velden,^37^^,^^38^ which expresses the extent of periodontal disease by considering the number of affected teeth and the severity of disease based on bone and attachment loss. Only patients with adult periodontitis (≥36 years) were considered.

#### Diabetic status

All patients completed an extensive health questionnaire, which included DM status (answered binary as yes or no), at intake. It was standard procedure for the periodontist to verbally confirm positive responses on the medical history document. As quality indicator, the DM patients were listed, and the prevalence was calculated as previously outlined in an earlier publication.^39^ For this analysis, the DM cases were defined prior to further data extraction. No distinction was made between type I and II DM. Patients with an unclear DM status were excluded.

#### Case and control selection

Each DM case was matched to an NDM control to evaluate the differences between groups. The matching procedure was performed by two researchers (MK and LPMW) in the following order: gender, year of birth, in agreement with Paljević et al (2024)^14^ and Poyato-Borrego et al (2020)^40^ with in addition matching based on the year of intake. Matching was continued until every DM case had a corresponding eligible control match. Age variations of one year were allowed if matched pairs were not born in the same year or visited the clinic for the first time in different calendar years. When more than one matched NDM control was available, the individual whose date of birth most closely approximated that of the DM subject was chosen.

DM patients and their matched controls were deemed suitable and selected for inclusion based on the following inclusion criteria:

• Aged 36 years or older.
• Diagnosed with moderate to severe adult periodontitis based on the criteria established by van der Velden.^38^
• Availability of comprehensive clinical records and radiographic data, including full-mouth radiographs, for thorough evaluation.
• Completion of a detailed health questionnaire, including self-reported DM status.
• Signed informed consent permitting the use of medical records for research purposes.

### Data extraction

#### Procedure

Of the selected DM and NDM cases, patient record files were manually reviewed, and data were extracted individually by two researchers (MK and LPMW). Any disagreement was resolved through discussion and consensus. A third reviewer (GAW) was consulted if required, and his judgment was considered to be decisive. Data extraction was performed by utilizing a custom-designed, standardized data extraction form. Extracted data were entered into a Microsoft Excel file and saved and could only be accessed with a password known to the research team.

The following parameters were extracted from eligible patient records: gender, age, year of periodontal intake, periodontal diagnosis, DM status, number of endodontically treated teeth, number of teeth, percentage of teeth and sites with probing pockets (PPD) >5 mm, and smoking behaviour. To ensure complete anonymization, any data associated with individual patients was deleted from the final dataset after the extraction was complete. When clinical data for a DM case were incomplete or missing, the case and NDM match were excluded from analysis. If information was incomplete or missing for an NDM case, the case was excluded from analysis, and a new match for the DM case was sought. This approach was also applied for incomplete and missing radiographic data.

#### Radiographs

Patients with a full set of dental radiographs were included. A set was deemed complete if peri-apical radiographs of all teeth were present or if a panoramic radiograph was available. A timeframe of one year was allowed between radiographs and the time of the periodontal intake clinical examination.

#### Number of teeth and endodontic treatment

The number of teeth and endodontically treated teeth were scored on radiographs and categorized according to type: molars, premolars, or anterior teeth (canines and incisors) and subdivided into upper and lower jaw. Teeth that contained any form of root-canal obturation were assessed as endodontically treated. Pulpotomised teeth, which retain vital pulp tissue in the root canals and do not undergo complete root canal obturation, were not classified as endodontically treated. Additionally, the number of teeth was extracted from records based on the periodontal chart to ensure the correct number of teeth were assessed.

#### Probing pocket depth (PPD)

In order to obtain a measure of periodontitis severity records from the periodontal intake appointments were reviewed for periodontal status. For this purpose, data on the number of teeth and total number of sites with PPD of >5 mm^41^ were extracted.

#### Smoking habits

Smoking habits were recorded from the medical questionnaire file and divided into three categories: current smoker, former smoker, and non-smoker. The number of cigarettes smoked per day was also recorded.

### Analysis

The anonymized raw data set in Excel (Microsoft Corporation) was transferred to the statistical analysis computer package, IBM SPSS Statistics 27 (Armonk, USA). Means, standard deviation, and frequency were used to generally describe the obtained data.

First, parametric tests were performed. Case and control subjects were directly compared using independent t-tests and χ^2^ (Chi-square) tests to assess possible variances between the groups demonstrated by 95% confidence intervals (CI), and *P*-values by difference between groups. Characteristics such as gender, age, and smoking habits were analysed. Additionally, analysis was performed for the following variables: number of teeth, percentage of endodontically treated teeth, percentage of teeth with PPD >5 mm, and percentage of sites with PPD >5 mm.^41^ Percentages were calculated to correct for the total number of teeth for each individual. It was decided ‘*a priori’* to perform sub-analyses for the number of endodontically treated teeth per tooth type and jaw type.

Second, multivariate logistic regression analyses were performed using a series of independent variables (DM, age, smoking, and number of teeth) to find the best fitting subset of risk indicators that predict the dependent variable: endodontically treated teeth. Odds ratios (OR) with 95% CI were calculated. For significant findings, the OR were interpreted according to Chen et al (2010)^42^ as equivalent to Cohen's d. Less than 1.68 was interpreted as no effect, ≥1.68 as small effect, ≥3.47 as medium effect, and ≥6.71 as large effect. Overall, the level of significance was set at *P* ≤ .05.

### Post hoc power analysis

‘Post hoc’ power analysis will be performed using statistical software (G power v.2.0, Bonn, Germany) to confirm based on the sample size the study power. A minimum of 80% power was considered necessary to confirm that the sample size was adequate for detecting statistically significant differences.

## Results

### Characteristics

In total, 344 DM visited the clinic between 2003 and 2019, of which 111 were not eligible to be included in this study according to the predefined criteria. The most common reason for exclusion was lack of full mouth radiographic information. Another common reason for exclusion was incomplete DM status. The 233 included periodontitis patients with DM were matched with 233 eligible periodontitis patients without diabetes, resulting in 466 cases and controls eligible for statistical analysis. The subjects ranged from 42 to 87 years of age, with a mean age of 65 years. In total, 54.9% of the subjects were male and 45.1% were female in the DM group, while, in the NDM group, 53.6% were male and 46.4% were female. In the DM group, 57% were smokers or former smokers. This was the case for 60% of the NDM group. The mean number of cigarettes for those that still smoked was 12.76 per day for the DM and 13.13 for the NDM. Analysis of the demographics showed no statistically significant difference resulting from proper matching.

### Severity of periodontitis

The mean percentage of teeth with a pocket depth >5 mm at patient level was 40% for DM and 39% for NDM (*P =* .64). The mean percentage sites with pockets >5 mm at patient level was 16% and 15%, respectively. There was no significant difference between the groups in the percentage teeth (*P =* .64) or sites (*P =* .68).

### Number of (endodontically) treated teeth

The mean number of remaining teeth was 25.2 (4.03) for the DM and 25.4 (3.94) for the NDM controls. Of the 233 diabetes patients, 153 had at least one endodontically treated tooth (66%), compared to 145 in the control group (62%).

The mean percentages of endodontically treated teeth were 6.9% (9.23) and 7.3% (9.62) for DM and NDM respectively. There was no significant difference between the groups regarding the mean number of teeth (*P* = .68) or the percentage of endodontically treated teeth (*P =* .60) (Table 2.1).

**Table 2.1:** Number of teeth, endodontically treated teeth, presented as mean and standard deviation of the included DM cases and their matched NDM controls.

	Mean (SD)		Statistical analysis		
N = 466	DM N = 233	NDM N = 233	Difference	95% CI	Test*
Mean number of teeth	25.20 (4.03)	25.35 (3.92)	0.15	[−0.57;0.88]	*P =* .68
Total number of teeth	5871 (4.03)	5907 (3.94)	36		
Mean number of endodontically treated teeth at patient level	1.65 (2.13)	1.80 (2.31)	0.15	[−0.26;0.55]	*P =* .48
Mean % endodontically treated teeth at patient level	6.88% (9.23)	7.34% (9.62)	0.87%	[−1.25;2.18]	*P =* .60

DM, diabetes mellitus; NDM, no diabetes mellitus; N, sample size; SD, standard deviation; CI, confidence interval.

⁎ Independent T-test.

### Sub-analysis distribution by jaw and tooth type

The sub-analysis of the number of endodontically treated teeth regarding the lower or upper jaw revealed no statistically significant differences between DM and NDM patients (95% CI: [−0.20;0.19] and *P =* .97, and 95% CI: [−0.12;0.42] and *P* = .28, respectively). To evaluate possible differences per type of tooth, an additional sub-analysis was performed. This subdivision by molars, premolars, and anterior teeth revealed no statistically significant differences between DM and NDM patients (Table 2.2).

**Table 2.2:** Sub-analysis of jaw and type of endodontically treated teeth on patient level presented as mean and SD of the included DM cases and the matched NDM controls.

	Mean (SD)		Statistical analysis		
N = 466	DM N = 233	NDM N = 233	Difference	95% CI	Test*
Maxilla (SD)	0.98 (1.44)	1.13 (1.56)	0.15	[−0.12;0.42]	*P =* .28
Mandibulae (SD)	0.67 (1.03)	0.67 (1.12)	0.00	[−0.20;0.19]	*P =* .97
Molars (SD)	0.74 (1.10)	0.77 (1.15)	0.03	[−0.18;0.23]	*P =* .81
Premolars (SD)	0.53 (0.94)	0.63 (1.08)	0.10	[−0.09;0.28]	*P =* .29
Anterior (SD)	0.38 (0.89)	0.40 (0.90)	0.02	[−0.14;0.18]	*P =* .80

DM, diabetes mellitus; NDM, no diabetes mellitus; N, sample size; SD, standard deviation; CI, confidence interval.

⁎ Independent T-test.

### Regression analysis

The continuous value of endodontically treated teeth was dichotomized and used for the regression model. When only DM was accounted for, the crude OR was 1.16 (95% CI: [0.80;1.70]; *P =* .44). Additionally, the analysis examined the association between endodontically treated teeth and DM-related factors, including age smoking status and number of teeth (see Online Appendix S4).

Table 3 summarizes the OR estimates and associated 95% confidence intervals. The OR does not change in the multiple linear regression analysis with correction for age, smoking and number of teeth. DM was not significantly associated with endodontically treated teeth (OR = 1.16;95% CI: [0.79;1.70]; *P =* .46).

**Table 3:** Multiple logistic regression on endodontically treated teeth.

Independent variables	OR (95% CI)	*P*-value
DM	1.156 [0.785;1.704]	.462
Age*	1.038 [1.017;1.059]	**<.001**
Total number of teeth	0.973 [0.922;1.027]	.324
Smoking (3 categories: non-smoker, former smoker, current smoker)	1.167 [0.922;1.478]	.199

DM, diabetes mellitus; CI, confidence interval; OR, odds ratio.

The bold values indicate statistically significant results (p < 0.05).

⁎ Continuous variable.

In addition, the effect of current and former smoking status and DM on the extent of endodontically treated teeth was explored using nonsmoking as a reference. The OR increased for current smokers (OR = 1.57) and showed a tendency towards statistical significance (*P =* .06) compared to people who never smoked. The OR being less than 1.68 this can be interpreted as no effect.^42^ For former smokers, this comparison resulted in an OR of 1.31 (*P =* .27) (Table 4).

**Table 4:** Multiple logistic regression on endodontically treated teeth with smoking as categorial variable.

Independent variables	OR (95% CI)	*P*-value
DM	1.175 [0.797;1.734]	.416
Age*	1.039 [1.018;1.061]	**<.001**
Total number of teeth	0.977 [0.925;1.031]	.396
Smoking Status Non-smoker⁎⁎ Current smoker Former smoker	1.565 [0.976;2.510] 1.306 [0.817;2.089]	.063 .265

DM, diabetes mellitus; CI, confidence interval; OR, odds ratio.

The bold values indicate statistically significant results (p < 0.05).

⁎ Continuous variable.

⁎⁎ Reference category

### Post hoc power analysis

The ‘post hoc’ power analysis estimated that a minimum inclusion of 65 subjects per group would achieve an 80% statistical power for the study.

## Discussion

DM is one of the most prevalent chronic medical conditions among dental patients.^43^ While the bidirectional link between DM and periodontal disease has been extensively discussed, less is known about its influence as a disease-modifying factor in the progression of caries lesions and the incidence of AP. This retrospective analysis aimed to evaluate the difference in the percentage of endodontically treated teeth between DM patients and NDM controls within a convenience sample of referred periodontitis patients. The prevalence of root-canal-filled teeth served as a surrogate for pulpal necrosis, which may result from extensive tooth decay. Our results indicated that in this population, DM was not a clinically relevant risk factor for the prevalence of endodontically treated teeth or tooth loss. The convenience sample used in this study was drawn from a private periodontal clinic, where full sets of patient radiographs were typically available for diagnostic purposes,^35^ allowing for a large sample with adequate radiographic information. All patients had been professionally diagnosed with periodontitis, and matching from the same patient population was considered an appropriate approach to compare groups with and without DM. Also, while periodontitis is linked to DM, it does not directly influence caries prevalence, suggesting that a sample of periodontitis patients does not inherently bias results related to caries experience.^35^

### DM as a potential risk factor for periapical disease

The possible role of DM as a risk factor for AP, in part as a surrogate for extensive tooth decay resulting in pulpal necrosis, was assessed by comparing the number of endodontically treated teeth between DM and NDM. No statistically significant differences were found regarding the percentage of endodontically treated teeth, the number of endodontically treated teeth per jaw or tooth type, or the number of teeth in general. This observation is supported by similar findings from a Brazilian non-periodontal patient sample in which an average of 2.8 and 3.4 for endodontically treated teeth were found in DM and NDM respectively (*P >* .05).^44^ However, in this Brazilian population AP was significantly more prevalent in untreated teeth from type II DM (15% versus 12% in NDM controls). Two prospective studies that have compared the outcome of root canal treatment in DM and NDM supported this supposition.^45^^,^^46^ It is therefore presumed that DM cannot be ruled out as a disease modifier of AP.

### Diabetes and tooth decay

The literature provides little or no consistent information on the role of DM as an etiological factor for tooth decay. It has been suggested that uncontrolled DM may result in an increased prevalence of dental caries.^47^ However, a SR found the literature does not describe a consistent relationship between type I DM and dental caries.^48^ This is supported by a recent scoping review concluding there is limited clinical evidence to support a clear relationship between hyperglycaemia and dental caries in humans.^49^ In contrast, long-term hyperglycaemia was found to induce dental caries in type I and type II diabetic rodents.^49^

### Diabetes and periapical radiolucencies

The prevalence of periapical radiolucencies has been widely studied. In Europe, it is estimated that periapical radiolucencies affect 61% of individuals and 14% of teeth, with prevalence increasing with age.^50^ These radiolucencies can be indicative of various conditions, including AP. Globally, the prevalence of AP is estimated at 52% at the individual level and 5% at the tooth level.^51^ Studies specifically investigating the relationship between DM and periapical radiolucencies suggest that DM may negatively impact endodontic outcomes and healing.^19^^,^^20^^,^^52^ A retrospective analysis of a Spanish university sample (70 subjects) found a significantly higher prevalence of periapical radiolucencies in patients with type II DM compared to non-diabetic controls.^53^ Another cross-sectional Spanish study (100 subjects) reported that individuals with DM had a significantly higher likelihood of having at least one root-filled tooth compared to NDM controls (70% vs. 50%).^30^

A recent systematic review concluded that DM increases inflammation and degeneration, particularly after dental procedures.^18^ An increased inflammatory state in hyperglycaemia could result in diminished healing of apical tissues. Based on these findings, DM has been suggested as a negative factor regarding the success of endodontic treatment^19^^,^^20^. A significant association has been shown between the presence of periapical radiolucencies in root-filled teeth among diabetics, reaffirming the potential impact of DM on endodontic healing.^15^^,^^44^ However, as the purpose of the present retrospective case-control analysis was to assess the percentage of endodontically treated teeth as the outcome parameter, no attempt was made to evaluate the presence of periapical radiolucencies or the success of endodontic treatment. Overall analyses and sub-analyses found that, in this periodontitis population, the prevalence of root-filled teeth among DM patients was not higher than among NDM controls.

### Diabetes and tooth loss

Endodontic therapy is a well-established intervention^16^ to preserve teeth that would otherwise require extraction due to damage or infection.^54^^,^^55^ It is generally a predictable treatment, resulting in a retention rate of up to 97% for treated teeth. However, approximately 3% of endodontically treated teeth require further treatment, most often resulting in extraction.^56^ The main reason for extraction is primarily non-restorable carious destruction.^57^

A recent SR that used the DMF index to assess caries experience found higher DMF index scores in DM patients compared to NDM.^8^ In the current study population, the number of remaining teeth between the DM and NDM controls with periodontitis was not significantly different. Another retrospective analysis reported comparable findings, concluding that there was no significant difference in the total number of teeth between these two groups, with an average of 21.7 teeth in DM patients and 22.8 in NDM controls (*P >* .05).^44^ These findings contrast with a recent SR which found that DM patients had a significantly higher risk of tooth loss.^11^ However, the reported risk ratio was found to be 1.63, which, although while statistically significant, was interpreted as a ‘small effect’.^58^ Moreover, it is poorly controlled DM that poses a particularly significant risk factor for tooth loss.^11^ Since DM control status could not be incorporated into the present retrospective analysis no distinction and sub-analysis between well-controlled and poorly controlled DM could be made.

### Diabetes and periodontitis

The relationship between periodontal disease and DM is a subject many authors have attempted to define. According to a Cochrane library review, the number of studies conducted on the link between DM and periodontitis has increased significantly in the past decade.^59^ In 2013, a consensus report on DM and periodontal disease was published by the European Federation for Periodontology and American Academy for Periodontology.^4^ This report reviewed epidemiological evidence from cross-sectional, prospective, and intervention studies on the role of periodontitis in DM and the underlying mechanisms. Periodontitis is proposed to adversely affect glycaemic control in DM. Moreover, a direct relationship was described between the severity of periodontal disease and DM-related complications.^4^ Although the findings with respect to periodontal disease severity (See Table 1) were not the primary purpose of this retrospective analysis, the mean percentage of periodontal pockets >5 mm was used as an indicator for the extent of periodontitis. In this study, the percentage of pockets >5 mm was found to be similar between DM and their matched controls. The present findings when considering pocket depth >5 mm as indicator for disease severity therefore do not support the supposition that periodontal conditions are significantly worse in DM periodontitis patients compared to NDM periodontitis controls.

**Table 1:** Characteristics of the included periodontitis patients and separated by matched DM status.

	Patients			Statistical analysis	
	DM N = 233	NDM N = 233	Total N = 466	Difference DM/NDM	Test
Sex Male (%) Female (%)	128 (54.9%) 105 (45.1%)	125 (53.6%) 108 (46.4%)	253 213	3 −3	0.78*
Age in years Mean (SD) Range	65.12 (10.15) 42-86	65.23 (10.06) 42-87	65 42 - 87	−0.09	*P =* .90 95% CI: [−1.96;1.72]†
Smoking status No smoker (%) Current smoker (%) Former smoker (%)	100 (42.9%) 62 (26.6%) 71 (30.5%)	94 (40.3%) 75 (32.2%) 64 (27.5%)	194 137 135	6 (2.6%) −13 (5.9%) 7 (3.0%)	0.41*
Smoking status No smoker/former smoker Current smoker	174 59	158 75	331 135	15 −15	0.13*
Mean number of cigarettes/day	12.76 (8.39)	13.13 (7.31)		−0.37	*P =* .79 95% CI: [−2.32;3.06]⁎⁎
Patients with an endodontically treated tooth (%)	153 (66%)	145 (62%)	298	8	0.44*
Severity of periodontitis (patient level)					
Mean % of teeth with pocket depth >5 mm	40.08% (24.80)	38.92% (28.88)		1.16%	*P =* .64 95% CI: [−6.06;3.74]
Mean % sites with pocket depth >5 mm	15.91% (13.17)	15.39% (14.11)		0.53%	*P =* .68 95% CI: [−3.01;1.96]

DM, diabetes mellitus; NDM, no diabetes mellitus; N, sample size; SD, standard deviation; CI, confidence interval.

⁎ Chi^2^ test.

† Independent T-test.

The present study retrospectively analysed data from patient records that involved periodontal treatment in the period of 2003‐2019. At that time, patients were classified at intake according to the definition of Van der Velden.^38^ Based on age, those patients that were classified as ‘adult periodontitis’ were selected for the present evaluation. Given the new classification^60^ which was more recently introduced patients would now be classified as having ‘periodontitis’ Therefore, it is difficult to compare this study direct to other studies that involve periodontal disease after 2018.

### Periodontitis and apical periodontitis

A histological study examining caries-free teeth with varying degrees of periodontitis demonstrated that pathological changes may occur in the pulp when periodontal disease is present.^61^ However, the pulp remains viable as long as the apical foramen is not compromised, suggesting that periodontal disease rarely jeopardizes the vital functions of the pulp unless it progresses to a terminal stage involving the primary pulpal blood supply.^62^ Clinical studies, however, highlight a reverse association between periodontitis and endodontic pathology. In patients with periodontitis, the marginal bone level has been correlated with both the percentage of root-filled teeth and the percentage of root-filled teeth with AP.^63^ A significant relationship was observed between periapical pathology and vertical bony defects, with teeth exhibiting periapical pathology showing significantly deeper periodontal pockets compared to those without. In mandibular molars with periapical lesions, significantly greater mean periodontal probing depths have been reported compared to teeth without such lesions, alongside a higher frequency of horizontal furcation involvement.^64^ Teeth with progressing periapical pathology demonstrated greater radiographic attachment loss than those without periapical pathology. Notably, an approximate threefold increase in the rate of marginal proximal radiographic bone loss was observed.^65^ These findings underscore the potential for AP, evident as periapical radiolucencies, to exacerbate periodontitis progression,^66^ likely through the spread of endodontic pathogens via patent accessory canals and dentinal tubules.

### Smoking and endodontic treatment

While smoking is considered as a risk factor for periodontitis, studies have shown no difference between smokers and non-smokers in terms of AP.^67^ In the present analysis the OR (1.57) for current smokers was close to being significant (*P =* .06) regarding the number of root-filled teeth. A cohort study conducted at the University of Basel, which included full-mouth periapical radiographs of 161 subjects (66 current smokers, 28 former smokers, and 67 individuals who had never smoked), found that smoking status did not predict AP in either females or males within this sample group.^68^ However, another study in a Portuguese population demonstrated that smoking increased the probability of developing AP.^69^ In addition, an SR^70^ found with moderate certainty that smokers had twice the chance of having a root canal treatment compared to nonsmokers (OR = 2.42). This supports the idea that smoking may be a contributing factor to the need for endodontic treatment. These contrasting findings highlight the controversial nature of the association between smoking habits and endodontic infection.^50^ It is also shown that both DM and smoking can impair the non-specific immune system and alter pulp and periapical healing after endodontic treatment.^50^ In the present analysis, no attempt was made to evaluate the success of the endodontic treatment. Nonetheless, it can be hypothesized that tobacco smoking is a negative prognostic factor for the outcome of root canal treatment, based on the outcome of a SR conducted in 2020.^71^ However, the overall strength of evidence was found to be low.

### Strengths and limitations

As the most important strength of this study, the distribution of age and gender was well-matched for the DM and NDM controls. Additionally, the population size of over 450 cases and controls contributes to the robustness and statistical power of the study. As part of the data extraction, the percentage of pockets >5 mm and smoking status of the included subjects were recorded. No difference between the two studied groups could be found. With this approach, the potential confounding factors were excluded, and the most reliable and valid estimation of the difference in the number of endodontically treated teeth between DM patients and NDM controls could be analysed.

On the other hand, this retrospective analysis had several limitations, as it did not differentiate between DM type I and type II. Previous research shows that DM types I and II interact differently regarding general health.^72^

Also, DM prevalence was assessed via self-report based on a medical history form and checked verbally by the periodontist at the intake appointment. Self-reported data are often argued to be unreliable and threatened by self-reporting bias.^73^, ^74^, ^75^ Moreover, it is possible the controls were not aware or did not report having DM: data from the National Health and Nutrition Examination Survey showed, based on laboratory results, undiagnosed DM was seen in 3.4% of all US adults and they represented 23% of all adults with DM.^76^

The study was done on a cohort of patients who all had periodontitis. The presence of periodontal disease may have introduced a potential confounding factor that could influence the association between DM and the prevalence of endodontically treated teeth. This may limit the generalizability of the findings to non-periodontitis patients.

Although key confounding factors such as age and smoking status were controlled for in the analysis, it is recognized that other variables – such as oral hygiene and dietary habits could further refine the understanding of the relationship between DM and oral health. These additional factors were not available in the patient records and, therefore, were not included in the analysis.

Lastly, it remains unclear whether patients were diagnosed with DM prior to experiencing tooth loss or extensive tooth decay, which has resulted in endodontic pathology. In addition, some instances of deep caries may lead to placement of deep restorations or crowns without endodontic treatment.

### Possible alternative reasons for our findings

Several factors could explain the result that the prevalence of root-filled teeth in DM patients was not higher than in NDM controls. One possibility is that DM patients may undergo root canal treatment more frequently but also experience a higher rate of subsequent tooth extraction, leading to an underrepresentation of root-filled teeth in our sample. As noted by Cabanillas-Balsera et al (2019),^71^ the risk of extraction was found to be more than twice as high for DM patients. Since our analysis only included existing teeth, previously root-filled teeth that were extracted would not have been accounted for. Additionally, differences in study populations may contribute to the discrepancy between our findings and previous studies^29^^,^^30^ reporting an elevated number of root-filled teeth in DM patients. Our sample consisted of referred periodontitis patients, who are already at an increased risk of tooth loss due to periodontal disease, which could have influenced the likelihood of retaining or extracting endodontically treated teeth. Furthermore, variations in treatment-seeking behaviour, dental care accessibility, and DM control may also play a role. Poorly controlled DM has been associated with delayed healing and an increased risk of post-treatment complications, potentially leading to a greater need for extraction.^77^ However, as we were unable to incorporate DM control status into our analysis, no distinction between well-controlled and poorly controlled DM could be made. These factors highlight the complexity of the relationship between DM, endodontic treatment, and tooth retention, warranting further investigation.

### Recommendations for further research

In future studies on DM versus NDM, incorporating actual HbA1c values, the DM type, the moment of DM diagnosis, and patient status could inform on DM type as an etiological factor in the prevalence of endodontic treatment. In future analysis, it would also be desirable to consider the distinction between the two types of DM and their status. Based on a recent SR, there is moderate certainty for a higher DMF index score in DM patients compared to those without DM.^8^ Difference between DM types may be the cause for apical pathophysiology resulting from extensive caries lesions reaching into the pulp,^48^^,^^78^ which would be an interesting line of further research. Additionally, future studies may control for a broader range of confounders to refine the understanding of the relationship between DM and the number of endodontically treated teeth.

## Conclusion

In this matched patient sample, diagnosed with adult periodontitis, there was no significant association between DM and the number of endodontically treated teeth or loss of teeth.

## Conflict of interest

The authors declare that they have no conflicts of interest.

## Appendix Supplementary materials

Image, application 1

## Footnotes

Supplementary material associated with this article can be found in the online version at doi:10.1016/j.identj.2025.100846.
